# Emerging Roles of Extracellular Vesicle-Delivered Circular RNAs in Atherosclerosis

**DOI:** 10.3389/fcell.2022.804247

**Published:** 2022-04-04

**Authors:** Cheng Wen, Bowei Li, Lei Nie, Ling Mao, Yuanpeng Xia

**Affiliations:** Department of Neurology, Union Hospital, Tongji Medical College, Huazhong University of Science and Technology, Wuhan, China

**Keywords:** atherosclerosis, extracellular vesicle, circular RNA (circRNA), Biomarker (BM), therapy

## Abstract

Atherosclerosis (AS) is universally defined as chronic vascular inflammation induced by dyslipidaemia, obesity, hypertension, diabetes and other risk factors. Extracellular vesicles as information transmitters regulate intracellular interactions and their important cargo circular RNAs are involved in the pathological process of AS. In this review, we summarize the current data to elucidate the emerging roles of extracellular vesicle-derived circular RNAs (EV-circRNAs) in AS and the mechanism by which EV-circRNAs affect the development of AS. Additionally, we discuss their vital role in the progression from risk factors to AS and highlight their great potential for use as diagnostic biomarkers of and novel therapeutic strategies for AS.

## Introduction

Atherosclerosis (AS) is a major cause of vascular death and results in ischaemic cardio-cerebrovascular disease and peripheral arterial disease worldwide (Herrington et al., 2016). Multiple risk factors, including but not limited to dyslipidaemia, diabetes, obesity and hypertension, were identified in early studies. Emerging evidence indicates that chronic infection plays a critical role in the pathological progression of AS (Pothineni et al., 2017). The continual influence of blood lipids and glucose, high vascular pressure and infectious agents leads to endothelial cells (ECs) dysfunction, chemotactic movement of monocytes towards vascular vessel walls, foam cell formation and phenotypic changes in smooth muscle cells (SMCs), eventually causing AS initiation and progression.

**FIGURE 1:**
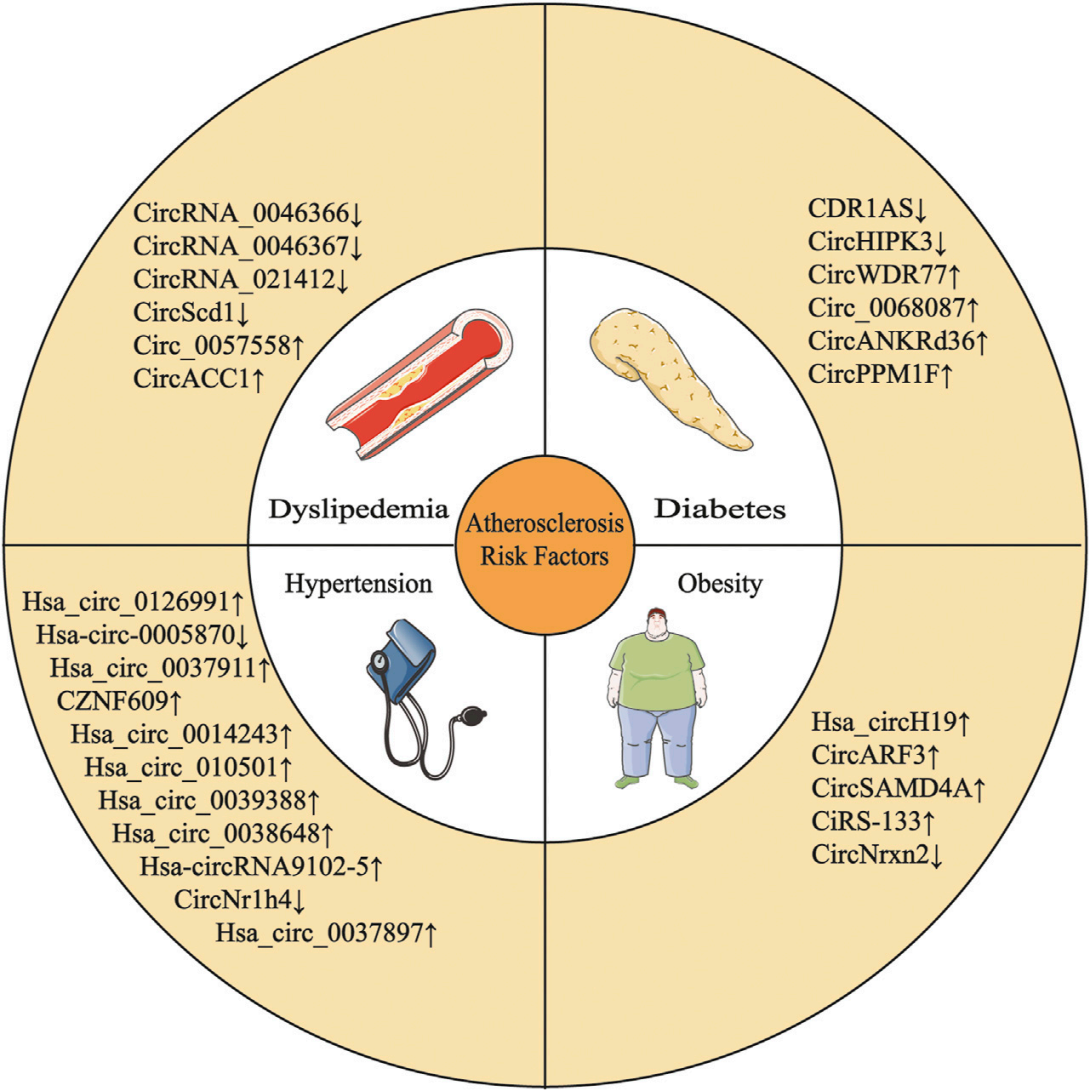
Multiple circRNAs are involved in the development of AS risk factors. ↑ : upregulation; ↓ : downregulation.

**FIGURE 2:**
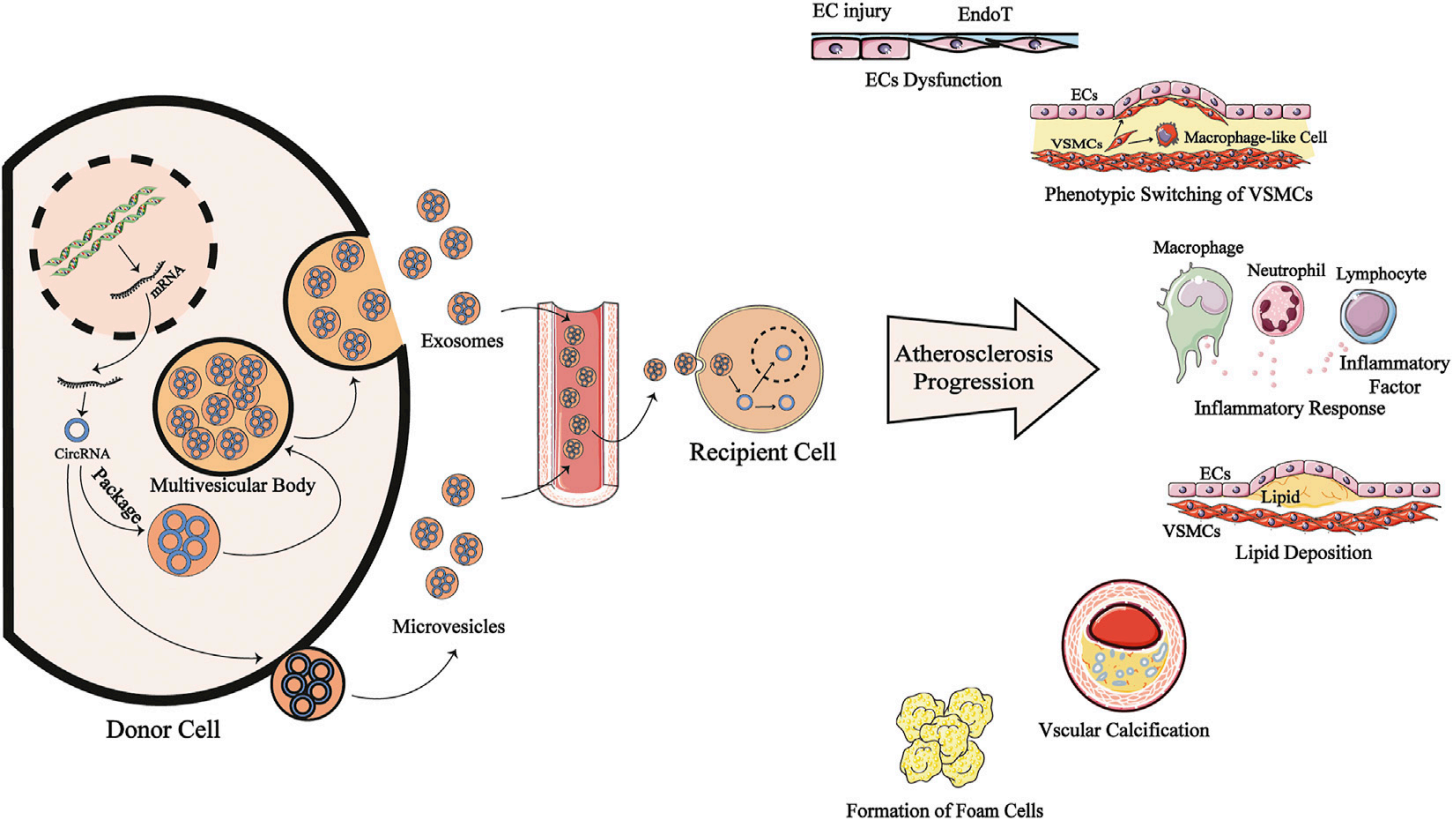
EV-transmitted circRNAs are involved in the progression of AS. CircRNAs are produced by back-splicing from mRNA and put into extracellular vesicles mainly exosomes and microvesicles, becoming EV-circRNAs. This process may be interfered by risk factors of antheroslerosis. After entering systemic circulation, theses EV-circRNAs are transported to recipient cells and regulating cells function in cytoplasm or nucleus. They may modulate the dysfunction of endothelial cells, phenotype switching of VSMCs, inflammatory response, lipid deposition, vascular calcification and the formation of form cells to participate in antherosclerosis pathogenesis. CircRNA: circular RNA; mRNA: messenger RNA; ECs: endotherlial cells; VSMCs: vascular smooth muscle cells; EndoT; Endothelial mesenchymal transition.

Circular RNAs (circRNAs) are a novel type of noncoding RNAs produced by back-splicing, which is a noncanonical splicing event. CircRNAs have a covalently closed single-stranded structure that is resistant to degradation by exonucleases (Kristensen et al., 2019). Initially, circRNAs were regarded as “evolutionary junk” originating from aberrant splicing events (Cocquerelle et al., 1993). However, in the past few years, the development of high-throughput sequencing techniques and circRNA-specific bioinformatics algorithms has contributed to the discovery of vast quantities of circRNAs. Studies have identified circRNAs expressed in cell-type- and tissue-specific patterns (Salzman et al., 2013; Szabo et al., 2015). CircRNA expression profiles associated with features of human tissues and signs of multiple diseases have been identified, and significant differences in these profiles indicate that circRNAs are involved in the pathological process of various disease (Figure 1 and Table 1), such as cardiovascular disease (Yu et al., 2020a), neurodegenerative disease (Li et al., 2020) and cancer (Fu et al., 2018). Qidong Cao et al. (2020) revealed a strong link between circRNAs and AS by summarizing the molecular mechanism by which circRNAs function in ECs, macrophages and vascular SMCs (VSMCs) in AS (Figure 2 and Table 2).

**TABLE 1:** CircRNAs are involved in the development of AS risk factors.

Risk factor	CircRNA	Dysregulation	Function	Cell/Model	Ref
Dyslipedemia	CircRNA_0046367	Down	Prevent hepatoxicity of lipid peroxidation against hepatic steatosis	HepG2 cells	Guo et al. (2017a)
	CircRNA_0046366	Down	Facilitate the transcriptional activation of lipid metabolism-associated genes	HepG2 cells	Guo et al. (2018)
	CircRNA_021412	Down	Disturb the balance between catalytic separation and adipogenesis	HepG2 cells	Guo et al. (2017b)
	CircScd1	Down	Impede lipid droplet formation and triglyceride content	AML-12 cells	Li et al. (2019a)
	Circ_0057558	Up	Facillitate lipogenesis and TG secretion	Huh-7 and HepG2 cells	Chen et al. (2021)
	CircACC1	Up	Facilitate the stability and activity of AMPK	HCT116 and LO2 cells	Li et al. (2019b)
	CDR1AS	Down	Increase insulin content and secretion	MIN6 cells	Xu et al. (2015)
Diabetes	CircHIPK3	Down	Impair the proliferation and capacity of insulin secretion and survival	Human islets cells	Cao et al. (2018)
	CircWDR77	Up	Promote high glucose induced proliferation and migration	Human VSMCs	Chen et al. (2017)
	Circ_0068087	Up	Promote chronic inflammation and vascular EC dysfunction in HG condition	HUVECs	Cheng et al. (2019)
	CircANKRd36	Up	Promote inflammation and cell apoptosis in the pancreatic tissues	T2DM rat model	Fang et al. (2018a)
	CircPPM1F	Up	promote activation and facilitate injury in pancreatic islets	PBMCs	Zhang et al. (2020a)
	Hsa_circH19	Up	Alleviate adipogenic differentiation	Human ADSCs	Zhu et al. (2020a)
Obesity	CircARF3	Up	Alleviate mitophagy-mediated inflammation and adipose inflammation	preadipocyte	Zhang et al. (2019a)
	CircSAMD4A	Up	Promote the differentiation of preadipocytes and obesity	preadipocyte	Liu et al. (2020)
	CircNrxn2	Down	Alleviate e WAT browning	preadipocyte	Zhang et al. (2019b)
	CiRS-133	Up	Promote WAT browning	preadipocyte	Zhang et al. (2019c)
	Has_circ_0126991	Up	Unknown	—	Liu et al. (2019a)
Hypertension	Has_circ_0005870	Down	Unknown	—	Wu et al. (2017)
	Has_circ_0037911	Up	Chang the concentration of Scr	—	Bao et al. (2018)
	CircZNF609	Up	Inhibit endothelial cell migration, tube formation, and apoptosis	HUVECs	Liu et al. (2017)
	Has_circ_0014243	Up	Unknown	—	Zheng et al. (2019)
	Hsa_circ_0105015	Up	Inflammatory pathways	HUVECs	He et al. (2021)
	Hsa_circ_0039388	Up	Enhance viability and invasive properties	HASMCs	Yin et al. (2020)
	Hsa_circ_0038648	Up	Enhance viability and invasive properties	HASMCs	Yin et al. (2020)
	Has-circRNA9102-5	Up	Unknown	—	Zheng et al. (2020)
	CircNr1h4	Down	Involved in hypertensive kidney injury	Mouse kidney collecting duct cells	Lu et al. (2020)
	Hsa_circ_0037897	Up	Unknown	—	Tao et al. (2021)

TG, total cholesterol; AMPK, AMP-activated protein kinase; EC, endothelial cell; HG, high glucose; WAT, white adipose tissue; Scr, serum creatinine; VSMCs, vascular smooth muscle cells; T2DM, type 2 diabetes mellitus; PBMCs, peripheral blood mononuclear cells; ADSCs, adipose-derived stem cells; HUVECs, human vein endothelial cells; HASMCs, human aortic endothelial cells.

**TABLE 2:** EV-circRNAs and their partners related to atherosclerosis.

CircRNA	Source	Recipient cell	Downstream pathways	Biological functions	Ref
CircIARS	Plasma	HMVECs	miR-122/RhoA	Reduce the permeability of the endothelial monolayer	Li et al. (2018)
Circ_0003204	Plasma	HUAECs	miR-370-3p/TGFβR2/Phosph-Smad3	Inhibit the proliferation, migration and tubular formation	Drew et al. (1981)
cPWWP2A	pericytes	HRVECs	miR-579–Angiopoietin1/Occludin/SIRT1	Affect the proliferation and migration of ECs and the formation of tubular structures	Wang et al. (2019a)
CircHIPK3	CMs	CMVECs	miR-29a/IGF-1	Inhibit the apoptosis of CMVECs and the production of ROS	Wen et al. (2021)
circRNA-0006896	Serum	HUVECs	miR-1264-DNMT1	Enhance proliferation and migration in HUVECs	Peng et al. (2021)
CircRNA-0077930	HUVECs	VSMCs	miR-622/Kras	Induce the VSMCs senescence	Chen et al. (2015)
CircDLGAP4	Plasma	Mouse brain ECs and MCs	miR-143/HECTD1	Inhibit EndoMT	Yang et al. (2018)
			miR-143/ERBB3/p-NFκB/MMP-2	Promote growth and fibrosis of MCs	Aimes and Quigley, (1995)

HMVECs, human microvascular endothelial cells; HRVECs, human retinal vascular endothelial cells; HUAECs, human atrial endothelial cells; CMs, cardiomyocytes; CMVECs, cardiac microvascular endothelial cells; HUVECs, human umbilical vein endothelial cells; MCs, mesangial cells; ROS, reactive oxygen species; EndoMT, endothelial-mesenchymal transition.

Extracellular vehicles (EVs), membrane-enclosed nanoscale particles derived from donor cells, carry proteins, RNAs and lipids to recipient cells and serve as intracellular communication mediators (Kim et al., 20172017). According to their size and biogenesis, EVs are classified into three types: exosomes, microvesicles and apoptotic bodies. Yang Li and his colleague first showed abundant and stable circRNAs in exosomes, and more than 1,000 circRNAs were found in human serum exosomes (Li et al., 2015). Some EV-circRNAs are even richer than their linear counterparts (Lasda and Parker, 2016). EVs are recognized and endocytosed by specific cells via specific interactions between surface membrane proteins, enabling the EV-circRNAs characteristic of tissue-specific regulation (Pegtel and Gould, 2019). Many studies have recently reported that EV-circRNAs are released into the microenvironment and participate in the metastasis, invasion and migration of tumour cells (Seimiya et al., 2020). Additionally, these circRNAs are closely related to AS, as indicated by their influence on EC function, VSMCs and macrophages as mediators of cell-to-cell communication.

In this review, we summarize the research progress of EV-circRNAs in AS and discuss their biological functions and significance in the pathogenesis of AS in the presence of risk or induction factors. In addition, we discuss the potential clinical applications of EV-circRNAs as biomarkers and treatment strategies in AS.

## Release of EV

EVs have been a research hotpot for a few years and some researchers have comprehensively reviewed the release mechanism of EVs. At present, it is generally believed that the formation of exosomes initiates early endosome formation by cell invagination. Then, under the regulation of the endosomal transport complex and some related proteins, early endosomes bud into the lumen of multivesicular bodies (MVBs). MVBs fuse with the cell membrane and secrete intracavitary vesicles called exosomes through the regulation of RAB enzymes in the GTPase family. Microvesicles, in contrast, are formed directly by budding and shedding of the plasma membrane (van Niel et al., 2018; Thietart and Rautou, 2020).

Stress conditions induce the release of EVs. *In vitro* tests have validated that shear stress, high glucose and the concentration of oxidatively modified low-density lipoprotein (oxLDL), TNFα and calcium can increase the exosome level. Thus, proatherogenic stimulation is strongly associated with the release of EVs (Giró et al., 2021).

## EV-CircRNAs are Involved in AS Risk Factors

### CircRNAs are Involved in AS Risk Factors

#### Dyslipidaemia

In recent years, circRNAs have been reported to participate in lipid metabolism. It has been found that in HepG2 cells treated with a high-fat diet (HFD), circRNA_0046366 promotes the accumulation of PPARα protein, which is translocated into the nucleus where it facilitates the transcriptional activation of lipid metabolism-associated genes (Guo et al., 2017a; Guo et al., 2018). Abnormalities in circRNA_021412/miR-1972/LPIN1 signalling lead to hepatic steatosis via the imbalance between catalytic separation and adipogenesis (Guo et al., 2017b). CircScd1 impedes lipid droplet formation and triglyceride (TG) content via the JAK2/STAT5 pathway in a HFD-fed mouse model (Li et al., 2019a). Overexpression of circ_0057558 positively correlates with TGs and modulates lipid metabolism in prostate cancer and facilitates lipogenesis and TG secretion in nonalcoholic fatty liver disease (Xia et al., 2018; Chen et al., 2021). CircACC1 was demonstrated to facilitate the stability and activity of AMPK, the central regulatory protein in energy metabolism (Li et al., 2019b). Most of these studies on circRNAs in lipid metabolism were focused on hepatic steatosis and cancer metabolic reprogramming, and few studies were focused on the influence of the reverse cholesterol transition, an important pathological process in the development of AS.

#### Diabetes Mellitus

CDR1AS was the first circRNA found to be associated with diabetes mellitus (DM), and it is the most compelling. The study showed that CDR1AS increased insulin content and secretion through the CDR1AS/miR-7/Myrip/Pax6 axis; in contrast, a subsequent study showed that a reduction in miR-7 decreased insulin secretion, while the overall insulin content was unaffected, and it diminished β cell proliferation without stimulating apoptosis in islets of db/db mice (Xu et al., 2015; Stoll et al., 2018). Silencing circHIPK3 led to negative regulation of the proliferation and insulin secretion capacity and survival in β cells (Stoll et al., 2018). Interestingly, circHIPK3 was downregulated in primary human aortic endothelial cells (HAECs), and accelerated HAECs apoptosis (Cao et al., 2018). Both EC injury and VSMC proliferation participate in AS, and some circRNAs probably play an important role in the pathological progression of AS induced by hyperglycaemia. CircWDR77 was reported to be the most significantly upregulated transcript in VSMCs under high-glucose conditions compared to its level in control VSMCs (Chen et al., 2017). Hsa_circ_0068087 and circANKRd36 were found to be correlated with chronic inflammation and vascular EC dysfunction in DM (Fang et al., 2018a; Cheng et al., 2019). In recent years, researchers have collected peripheral blood or islet samples from DM patients and healthy controls and performed comprehensive comparisons with microarrays. Large amounts of circRNAs have been found to be remarkably differentially expressed in human α and β cells and blood cells (Kaur et al., 2018; Stoll et al., 2018; Yang et al., 2020a; Luo et al., 2021). Among these circRNAs, circPPM1F was upregulated in mononuclear cells in the peripheral blood of children with T1DM, and it promoted M1 macrophage activation and facilitated injury to pancreatic islets in diabetic mice by competitively interacting with HUR, inhibiting the expression of PPM1F (Zhang et al., 2020a).

#### Obesity

Substantial efforts have been devoted to analysing the mechanism by which circRNAs influence the development of obesity, such as the role of circRNAs in fat formation. Hsa_circH19 overexpression was found to protect against obesity, and interestingly, it was also upregulated in metabolic syndrome (MetS) and related to lipid-related parameters. The deletion of circH19 activated the transcription of lipogenic genes, leading to the differentiation of human adipose-derived stem cells (ADSCs) and buffering lipid flux (Zhu et al., 2020a). Excess adiposity induces the recruitment of immune cells, mainly macrophages, to fat depots, which release proinflammatory cytokines/chemokines that activate chronic and low-grade inflammation (Reilly and Saltiel, 2017). CircARF3 block the effects of miR-103, which result in the activation of TRAF3 expression, suppress the nuclear factor κB (NF-κB) pathway, exacerbate mitophagy and inhibit NLRP3 inflammasome activation, ultimately alleviating mitophagy-mediated inflammation in mouse adipose tissue (Zhang et al., 2019a). A new strategy was provided in this study to prevent adipose inflammation in obesity disorder. Obesity is partially attributed to genetic factors, and Cheng et al. illustrated that circRNAs contribute to lipogenesis and the development of chronic metabolic disease in the offspring of mothers with obesity, and they hypothesized that circRNAs may be involved in epigenetic regulation (Chen et al., 2020a). Studies have shown that circRNAs are closely related to adipogenesis and that their expression is dynamically regulated during adipogenesis (Sun et al., 2020). For example, circSAMD4A expression was significantly higher among differentially expressed circRNAs, and the circSAMD4A/miR-138-5p/EZH2 axis was proven to modulate the differentiation of preadipocytes in a mouse model (Liu et al., 2020). In addition to participating in regulating adipogenesis, circRNAs have been shown to influence the browning of white adipose tissue (WAT), which was found to be a novel method of obesity prevention because it promoted energy metabolism. Both ciRS-133 and circNrxn2 have been implicated in WAT browning. CiRS-133 in plasma exosomes obtained from gastric cancer patients stimulated WAT browning by activating PRDM16, and circNrxn2 had the same effect by increasing the expression levels of fibroblast growth factor 10 (FGF10) in mice fed a HFD (Zhang et al., 2019b; Zhang et al., 2019c).

#### Hypertension

Hypertension is a systemic disease, and its complex pathogenesis makes it difficult to elucidate the precise molecular mechanisms involved. Numerous studies have demonstrated that circRNAs are potential biomarkers and important modulators in hypertension (Liu et al., 2017; Wu et al., 2017; Bao et al., 2018; Liu et al., 2019a; Zheng et al., 2019; Lu et al., 2020; Yin et al., 2020; Zheng et al., 2020; He et al., 2021; Tao et al., 2021). CircRNA array analysis of blood samples obtained from patients with essential hypertension and healthy individuals showed that the expression of circRNAs was significantly different between these groups. These researchers found that high expression of circRNAs was accompanied by low expression of relevant miRNAs, and they hypothesized that circRNAs may participate in the development of hypertension by acting as miRNA sponges. The upregulation of hsa-circRNA9102-5 expression was accompanied by the downregulation of hsa-miR-150-5p expression (Zheng et al., 2020), which was proven to promote angiogenesis and EC migration *in vitro* and *in vivo* in vascular ECs (Li et al., 2013). In addition, hsa-circ-0105015 expression was upregulated and hsa-miR-637 expression was downregulated in HAECs and human vein ECs (HUVECs) treated with TNF-α, suggesting that these circRNAs may be associated with vascular inflammation and endothelial dysfunction in hypertension (Yao et al., 2021).

### EV-CircRNAs are Involved in Risk Factors for AS Formation and Development

EVs can act as biological vectors carrying nucleic acids, proteins and lipids through their interaction with recipient cells involved in the pathogenesis of human disease. Exosomes are effective mediators between key cells involved in diabetes and AS. It was reported that obesity changed the miRNA profile of circulating exosomes, inducing glucose intolerance and dyslipidaemia in lean mice (Castaño et al., 2018). EVs containing miR-221–3P from perivascular adipose tissue (PVAT) mediate vascular remodelling by inducing phenotypic switching of vascular cells and inducing inflammatory responses in PVAT (Zhu et al., 2019a). Visceral fat–derived exosomes, irrespective of obesity, stimulated foam cell formation from macrophages through the interference of mediated cholesterol efflux induced by ABCA1 and ABCG1 and the inhibition of M1 phenotype switching during atherogenesis (Xie et al., 2018). Under high-glucose conditions, cardiomyocytes of rats with type 2 DM (T2DM) released ‘harmful’ exosomes that impaired endothelial function. Cystatin C was proven to be a potentially useful marker in the early stage of AS in T2DM patients without obvious thickening of arterial wall, (Kaneko et al., 2018). A significant relation between EV-cystatin C and MetS was observed in patients with clinically manifested vascular disease (Kranendonk et al., 2014). High glucose levels induce an increase in S100A9, which was proven to accelerate the secretion and calcification-induction potential of EVs derived from primary macrophages (Xie et al., 2018). Small extracellular vesicles (sEVs), described as EVs originating from the endosomal compartment, are increased in patients with MetS (Hafiane and Daskalopoulou, 2018; Simon et al., 2018); in these patients, circulating sEVs may contribute to metabolic endotoxaemia, leading to endothelial dysfunction by delivering LPS and inducing TLR4-dependent ROS production (Ali et al., 2021). The hallmark of AS is low-grade vascular inflammation, and hyperglycaemia induces the activation of monocytes and ECs and the production of cytokines and adhesion molecules, such as intracellular adhesion molecule type-I (ICAM-1) (Altannavch et al., 2004). High glucose levels led to elevated concentrations of exosomes produced by ECs and monocytes. *In vivo*, these exosomes increased ICAM-1 expression in Mono-Mac-6 cell, monocyte and HUVEC models, suggesting that exosomes may be involved in the potential mechanism underlying inflammatory cell activation in DMT2 and in diabetic complications in cardiovascular system (Sáez et al., 2019).

Hypertension may impair the function of vascular components by changing the components of EVs. The level of miR-17, a negative regulator of ICAM1, was decreased in macrophage-derived exosomes obtained from rats with hypertension, and these exosomes elevated the expression levels of ICAM1 and PAI-1 in HCAECs. The study suggested that EC-related inflammation under hypertensive conditions is caused, at least in part, by macrophage-derived exosomes. The angiotensin-converting enzyme (ACE) content was increased and the miR-155-5p content was reduced in adventitial fibroblast-derived EVs obtained from spontaneously hypertensive rats (SHRs) but not in normotensive Wistar–Kyoto rats (WKRs). These SHR-derived EVs promoted the proliferation of both WKR-VSMCs and SHR-VSMCs and led to deterioration in vascular remodelling after intravenous injection of SHR-EVs in WKRs and SHRs (Ren et al., 2020).

## EV-CircRNAs in the Progression of AS

A crucial process in AS development is foam cell formation and accumulation within the lipid-rich subendothelial space of the affected artery (Bäck et al., 2019). Initially, the aforementioned proatherogenic factors lead to EC injury or even dysfunction (Zhu et al., 2018). Lipids in the blood enter the arterial wall, and monocytes are attracted to the area and then differentiate into macrophages. Activated macrophages phagocytose accumulating lipids and secrete chemokines and inflammatory cytokines (Tabas and Lichtman, 2017). With the development of chronic inflammation, VSMCs induce phenotypic changes, phagocytose lipids, and migrate to the subintima of arteries. Due to lipid metabolic pathway dysregulation, lipid-dense macrophages and VSMCs, called foam cells, eventually undergo apoptosis, leading to necrotic core formation. In the late stage of AS, extracellular matrix (ECM) proteins are degraded by inflammatory cytokines and matrix metalloproteinases (MMPs), resulting in plaque rupture, and leading to myocardial infarction (MI) or stroke (Doyle and Caplice, 2007). Increasing evidence has implicated EV-circRNAs in the pathogenesis of AS, and a comprehensive understanding of the functional mechanism of their roles will lay a solid foundation for the development of EV circRNA‐based diagnostic and therapeutic interventions in AS.

### EC Dysfunction

#### EC Injury

According to the injury response theory, AS is considered to be the result of chronic inflammation in the arterial wall that damages ECs. The initiating factor of AS is EC function disruption. Furthermore, EC damage causes leukocyte adhesion, vasoconstriction, platelet activation, oxidative stress and inflammation, thrombosis, and blood coagulation and ultimately leads to cardiovascular disease (Behrendt and Ganz, 2002).

Increasing evidence shows that noncoding RNAs carried in exosomes are involved in regulating the occurrence of EC dysfunction. CircIARS is elevated in plasma exosomes and tumour tissues from patients with pancreatic cancer, and it reverses the inhibition of the target gene RhoA by accelerating the adsorption of miR-122 onto human microvascular ECs, which reduces the permeability of the endothelial monolayer (Li et al., 2018). An atherosclerotic cell model generated by stimulants such as oxLDL, proinflammatory cytokines, advanced glycation end products and reactive oxygen species (ROS) verified that circRNAs were associated with the regulation of EC functions. CIRC_0003204 expression was downregulated in HAECs treated with oxLDL to inhibit the proliferation, migration and tubular formation of atherosclerotic ECs through the miR-370–33p/TGFβR2/p-Smad3 axis. CIRC_0003204 expression was found to be remarkably increased in plasma exosomes in patients with cerebral AS, suggesting that it may be transported from exosomes to vascular ECs, where it plays a role (Drew et al., 1981). The circRNA cPWWP2A regulates diabetic retinal microangiopathy by participating in the crosstalk between pericytes and ECs. cPWWP2A mainly exists in the cytoplasm of pericytes in the retina. Hyperglycemia increases the expression of cPWWP2A in these pericytes but does not affect ECs. It was confirmed that cPWWP2A was transported between pericytes and ECs by exosomes, affecting the proliferation and migration of ECs and the formation of tubular structures (Liu et al., 2019b). Exosomal circHIPK3 derived from cardiomyocytes preconditioned through hypoxia exposure was transferred to cardiac microvascular ECs (CMVECs) under oxidative stress conditions. CirHIPK3 inhibited the apoptosis of CMVECs and the production of ROS, providing new insights into the mechanism of microvascular dysfunction induced by hypoxia (Wang et al., 2019a). The Serum exosomal circRNAs expression profile of patients with stable plaque atherosclerosis (SA) and unstable/vulnerable plaque atherosclerosis (UA) was explored by circRNA array, and circRNA-0006896, upregulated both in SA plaque and HUVECs, significantly enhances proliferation and migration in HUVECs treated with serum exosomes of SA patients (Wen et al., 2021). Additionally, circ-USP36 was found to be elevated in serum samples of atherosclerotic patients and to promote the apoptosis and inflammation of HUVSMCs and HUVECs activated by ox-LDL (Peng et al., 2021). CircRNA ZNF609 was significantly upregulated in peripheral blood leukocytes in patients with coronary artery disease. It was verified through *in vitro* experiments that circRNA ZNF609 plays a protective role by inhibiting the release of proinflammatory factors and the apoptosis of HUVECs subjected to oxidative and hypoxic stress (Liu et al., 2017; Liang et al., 2020). Evidence suggests that microvesicles produced by ageing ECs in culture promote the calcification of VSMCs (Alique et al., 2017). In subsequent research, circRNA-0077930 in hyperglycaemia-stimulated vascular EC exosomes was found to induce the senescence of VSMCs and was closely related to cardiovascular disease in diabetic complications (Wang et al., 2020a).

An increasing number of studies have concentrated on the effect of circular RNAs on EC function. The mechanism by which exosomal-derived circRNAs affect ECs has not been fully explained. CircRNAs, which can affect the function of ECs and other cells, may be involved in the crosstalk and progression of AS *via* exosomal cargo transport.

#### Endothelial-Mesenchymal Transition

Endothelial-mesenchymal transition (EndoMT) is characterized by phenotypic changes in normal ECs manifesting by the acquisition of mesenchymal cell morphology and properties similar to those of fibroblasts and SMCs, including the promotion of proliferation, migration, expression of various leukocyte adhesion molecules, and secretion of ECM proteins such as fibronectin and collagen. EndoMT promotes the progression of AS (Chen et al., 2015), and endothelial lineage tracking showed that EndoMT is an important mechanism for the accumulation of activated fibroblasts and SMC-like cells in atherosclerotic plaques (Evrard et al., 2016). EndoMT is involved in the regulation of inflammation in the pathological process of AS. Inflammatory factors relieve FGFR1 inhibition of the TGFβ signalling pathway [TGF-β plays a central role in promoting the progression of EndoMT (Medici et al., 2010; van Meeteren and ten Dijke, 2012)] by inhibiting the expression of FGFR1, promoting the interstitial movement of ECs and promoting the expansion of plaques (Chen et al., 2015). In addition, EndoMT also affects the phenotype of unstable plaques. EndoMT-derived fibroblasts can affect the stability of atherosclerotic lesions by changing the balance of collagen and MMPs and by promoting the transition of stable plaques into collagen-deficient and easily ruptured plaques with thinner fibre caps (Evrard et al., 2016).

An increasing number of miRNAs have been confirmed to participate in EndoMT, and circRNAs have also been confirmed to function as sponging miRNAs to regulate EndoMT (Hulshoff et al., 2019). In rat coronary EndoMT induced by TGF-β1, 102 circRNAs were differentially expressed, 66 circRNAs were upregulated and 36 circRNAs were downregulated (Herrington et al., 2016). CircDLGAP4 acted as a miR-143 sponge to inhibit the expression of HECTD1, tight junction proteins and mesenchymal markers and to inhibit EndoMT (Bai et al., 2018). Li Yang et al. studied HVMECs *in vitro* and confirmed that circHECW2 inhibited its effect by sponging MIR30D and that MIR30D targeted ATG5 to inhibit the EndoMT of cells (Yang et al., 2018). Moreover, circHECTD1 inhibited the interstitial movement of mouse lung microvascular ECs induced by SiO2 by regulating the expression of its host gene, hectd1 (Fang et al., 2018b). Multiple exosomal circRNAs were reported to induce epithelial-mesenchymal transition (EMT) to promote the malignant phenotype and the metastasis and invasion of cancer cells (Chen et al., 2018a; Wang et al., 2020b; Zhu et al., 2020b; Wang et al., 2021a). EndoMT, a specialized form of EMT, has similar morphological and molecular characteristics to EMT and was shown to be modulated by exosomal circRNAs in recent studies. After being found in acute ischaemic stroke, circ_DLGAP4 was studied in exosomes. Exo-circ_DLGAP4 was increased in the serum of diabetic kidney patients and T2DM patients as well as in the culture medium of high glucose-treated mesangial cells (MCs). Circ_DLGAP4 promoted the growth and fibrosis of MCs by combining with miR-143 and then inhibiting the protein expression of ERBB3/p-NF-κB/MMP-2 (Bai et al., 2020a). Notably, activated NF-κB participates in inflammation-induced EndoMT because it is an important transcription factor of the FGF2 gene, a direct mediator of EndoMT (Bai et al., 2020a). Furthermore, some studies reported that increased expression of MMP-2 affected cell migration by degrading specific ECM components and exhibiting a mesenchymal phenotype (Aimes and Quigley, 1995; Giannelli et al., 1997; Song et al., 1999). According to these results, circ_DLGAP4 in plasma exosomes may play a protective role in pathophysiological progression by inhibiting EndoMT.

Although few studies have investigated how exosomal circRNAs modulate EndoMT in AS, multiple reports have indicated that exosomes are associated with cell-to-cell communication in EndoMT, especially in communication between ECs and macrophages. YING YANG et al. found that foam cells derived from M1 macrophages upregulated CCl-4 to induce EndoMT and accelerate the progression of AS by increasing endothelial permeability and monocyte adhesion, disrupting endothelial function (Yang et al., 2017). Alexandra et al. proved that ECs can acquire mesenchymal characteristics and an incomplete EndoMT phenotype through interaction with macrophages. Macrophages promote the expression of endothelial colony-stimulating factor, and conditioned medium from cells undergoing EndoMT reduces the proliferation and the expression of antigen-presenting cell markers and TNF-α in macrophages treated with ox-LDL (Helmke et al., 2019). Endothelial cells and macrophages play important roles in AS. Cells undergoing EndoMT interact with macrophages, but the molecular mechanism of this interaction remains to be studied. Evidence showing that exosomes and circular RNA participate in information transmission between ECs and macrophages may provide new ideas for potential therapy.

### Phenotypic Switching of VSMCs

In the process of AS phenotypic switching, VSMCs decrease the expression of a range of ‘SMC markers’ (including smooth muscle cell myosin heavy chain, SM22α/tagln, and smooth muscle cell actin) and increase the capacity for cell proliferation, migration and secretion of various extracellular matrix proteins and cytokines (Alexander and Owens, 2012; Bennett et al., 2016). This process has been considered of great importance to AS. EVs function as messengers among atherosclerotic-associated cells. Macrophage- and EC-derived EVs regulate phenotypic switching of VSMCs, which has been proven *in vitro*. Macrophage foam cell-derived EVs enhance VSMC migration and adhesion capacity, but the effective cargo is unknown for these changes (Niu et al., 2016). Noncoding RNAs have been found to be involved in this progression, with miR221, miR-222, miR-21-3p, microRNA-19b-3p, and LINC01005 mediating phenotypic modulation by activating synthetic genes or promoting VSMC proliferation and migration to accelerate AS development (Leung et al., 2013; Zhu et al., 2019a; Wang et al., 2019b; Zhang et al., 2020b; Wang et al., 2022a). microRNA-92a from EC-derived EVs has a similar functional mechanism to macrophage-derived EVs (Wang et al., 2022b). Interestingly, in obese mice fed a HFD, VSMCs can take up EVs derived from perivascular adipose tissue and their packaged miRNAs tested *in vivo* and *in vitro*, and miR-221-3p as a highly enriched miRNAs leads to vascular dysfunction by restraining the expression of contractile genes, provoking early-stage vascular remodelling under the obesity-associated inflammation conditions (Li et al., 2019c). These data further elucidate the mechanism of how obesity develops into vascular dysfunction in AS.

EV-circRNAs were first found to regulate VSMC function. Under high-glucose conditions, circRNA-0077930 is transported by HUVEC exosomes to induce the senescence of VSMCs in diabetic vascular complications (Wang et al., 2020a). However, other pathological changes in VSMCs have not been intensively studied. While multiple circRNAs have been reported to regulate VSMC phenotypic switching (Kong et al., 2019; Peng et al., 2020a; Sun et al., 2021; He et al., 2022), whether EV-circRNAs are involved in crosstalk among the three types of cells has not been sufficiently studied and few definite conclusions can be reached.

### Inflammatory Response

#### Inflammation Induced by Infection

Increasing evidence has indicated that infection is closely related to AS and that pathogens influence the progression of AS in a direct or indirect way. A large number of infectious agents were detected in AS plaques but not within normal blood vessels, including *Porphyromonas gingivalis* (Velsko et al., 2014), *Treponema denticola* (Chukkapalli et al., 2014), Hepatitis C virus (HCV) (Boddi et al., 2010), *Chlamydia pneumoniae* and *Helicobacter pylori* (HP) (Kaplan et al., 2006) and cytomegalovirus (Cao et al., 2017), which indicated a direct role for pathogens in local plaque development. Pathogen infection at nonvascular sites, such as C. pneumoniae located in the lung, HCV located in the liver and HP located in the gastric area, also accelerates AS indirectly by activating the immune system and inducing chronic low-grade inflammation. These pathogens ultimately promote AS by activating EC inflammatory responses, increasing macrophage-derived foam cell formation, promoting SMC proliferation and migration and inhibiting apoptosis.

In 1976, circular RNAs were first discovered in Sendai virus and plant viroids by electron microscopy (Kolakofsky, 1976; Sanger et al., 1976). Several circRNAs from virus or host cells were reported to be important in the biological behaviours of pathogens in infected cells. In 2018, Yue Zhang and his colleagues found that 1,365 circRNAs were differentially expressed between 3 HARRT-naive patients with early HIV-1 infection and 3 healthy controls, including 912 upregulated circRNAs and 453 downregulated circRNAs. These circRNAs were then found to be involved in the immune, inflammatory, and defence responses to viral infection, playing important roles in the pathogenesis and disease progression of HIV infection (Zhang et al., 2018a). CircPSDD3 regulated RNA amplification in a pro-viral manner during a post translational step by binding factor eIF4A3 and inhibiting the nonsense-mediated decay (NMD) pathway to enhance HCV RNA imbalances in infected liver cells. CircGATAD2A regulates H1N1 replication in VPS34-dependent autophagy in the A549 cell line (Yu et al., 2019). Jingui Deng et al. reported that human cytomegalovirus (HCMV) influenced host circRNA transcription and suggested multiple functions of circSP100 with multiple important HCMV-encoded proteins through circSP100-binding sites (Deng et al., 2021). In addition to interacting with viral proteins, circRNAs participate in P. gingivalis-induced periodontal inflammation. The circRNA CDR1as was downregulated in human periodontal ligament stem cells (PDLSCs) in the context of inflammation induced by P. gingivalis-derived lipopolysaccharide. CDR1as enhanced PDLSC proliferation through the CDR1as/miR-7/ERK pathway upon both overexpression and knockdown of CDR1as (Wang et al., 2019c). CircRasGEF1B, which has a human homologue with similar properties, upregulates the expression of ICAM-1, inducing LPS stimulation. This effect was proven to depend on the activation of NF-κB through the TLR/LPS pathway (Ng et al., 2016). This finding indicates that circRNAs may regulate the phenotypic transformation of monocytes in the innate response. According to these studies, increasing the abundance of infectious agents regulated by circRNAs may increase pathogen abundance in blood circulation, enhance systemic inflammation, promote pathogen invasion into blood vessel walls and activate ECs to induce serious atherosclerotic changes. CircRNAs seem to play irreplaceable roles in infection-associated AS.

Bacteria release membrane vesicles (BMVs), which have a structure similar to that of EVs and a distinct composition and content, play a pivotal role in the survival and replication of bacteria in infectious hosts by affecting virulence, horizontal gene transfer, export of cellular metabolites, phage infection and cell-to-cell communication (Mashburn and Whiteley, 2005; Toyofuku et al., 2019). Outer membrane vesicles (OMVs) primarily originate from Gram-negative bacteria. *P. gingivalis* participate in the pathological process of cardiovascular diseases such as AS and thromboembolism. A study indicated that OMV exposure led to increased runt-related transcription factor 2 (Runx2) expression and subsequently affected ERK signalling to promote VSMC calcification. However, the contributing OMV component is unknown (Yang et al., 2016). Bacteria also modulate the formation and components of EVs in infected host cells involved in infection-induced AS. Epidemiological and clinical studies showed a close correlation between infection with CagA-positive HP and a high incidence of AS and plaque instability compared to the effect of infection with CagA-negative HP (Pietroiusti et al., 2002; Zhang et al., 2008). In a recent study, serum-derived EVs carried CagA into the blood circulation in patients infected with CagA-positive HP (Shimoda et al., 2016). Further research revealed that CagA was packaged into exosomes derived from infected gastric epithelial cells and was taken up in aortic plaques, where it promoted AS by inducing macrophage foam cell formation (Yang et al., 2019). CagA-containing exosomes enter ECs and impair their function. Researchers used exosomes obtained from conditioned medium obtained from human gastric epithelial cell culture with CagA^+^ HP or serum exosomes derived from patients or mice with HP infection and proved that these exosomes significantly decreased EC functions, reducing EC migration, tube formation, and proliferation *in vitro* (Xia et al., 2020).

Viruses that infect cells may increase and/or alter exosomal content under pathological or stress conditions (de Jong et al., 2012), and this alteration may be the mechanism underlying the systemic response caused by local infection. In HIV-infected individuals, monocytes are activated into an IFNα phenotype, and the levels of circulating LPS are elevated. Exosomes from activated monocytes enter ECs and cause injury via the TLR4 and NF‐κB pathways, which may contribute to vascular disease in people with HIV infection and other diseases associated with chronic immune activation (Tang et al., 2016). D.R.A. Reis et al. (2020) hypothesized that human papilloma virus (HPV) infection can enhance systemic inflammation and modulate the release of nucleic acids carried by EVs directly targeting blood vessels, which may be the reason for the high risk of cardiovascular diseases in infected patients and may promote atheroma formation, and they discussed possible experimental approaches on the basis of extensive literature.

#### Proinflammatory Mechanism

EVs derived from steatotic hepatocytes accelerate endothelial inflammation and promote atherogenesis by suppressing KLF4 and activating NF-κB (Jiang et al., 2020). Exosomes derived from mature dendritic cells play a role similar to those of steatotic hepatocytes by triggering the NF-κB pathway (Gao et al., 2016). Large circulating EVs in MetS participate in enhanced SMC proliferation, migration, a proinflammatory profile, and activation of the ERK5/p38 pathway, leading to vascular inflammation and remodelling in AS. During inflammation, exosomes secreted by macrophage-transported microRNAs accelerate the development of AS (Nguyen et al., 2018; Fitzsimons et al., 2020). In addition, exosomes from platelets, vessel cells (He et al., 2018; Bai et al., 2020b), inflammatory adipocytes (Wadey et al., 2019) and dendritic cells increased the level of inflammatory factors and recruited inflammatory cells and promoted their adhesion to the vessel wall, leading to a chronic inflammatory response process in AS. Multiple circRNAs (circ_0004104 (Zhang et al., 2021a), circular ANRIL (Song et al., 2017), circTM7SF3 (Wang and Bai, 2021), circUSP36 (Miao et al., 2021) and so on) were upregulated in AS and demonstrated to induce vascular EC injury and oxidative stress and inflammation. Circ_GRN and Circ_CHFR were also upregulated in atherosclerotic serum and expedited the inflammation of human vascular muscle cells (Zhuang et al., 2020; Li et al., 2021a). The aforementioned diseases are closely linked to systemic inflammation and the induction of oxidative stress in cells indicating a possible mechanism by which these diseases change the contents of EVs transported during the pathogenesis of AS.

Orally administered *Lactobacillus* strains reportedly reduced inflammatory cytokine production in mice. The anti-inflammatory activity was partially mediated by circulating exosomes decreasing the production of TNF-α and IL-6 in macrophages (Aoki-Yoshida et al., 2017). Gingival mesenchymal stem cell-derived exosomes play important roles in periodontitis-related AS. They reduced the expression of inflammatory factors and the amount released, promoted the polarization of proinflammatory macrophages into an anti-inflammatory phenotype, and inhibited lipid accumulation in a high-lipid microenvironment (Zhang et al., 2021b). Exosomes secreted by naive bone marrow-derived macrophages (BMDM-EXOs) transmit anti-inflammatory microRNAs to recipient macrophages, which foster M2 polarization by targeting NF-κB and TNF-α signalling (Bouchareychas et al., 2020). T helper (Th17) cells were shown to promote AS in their role as a novel subset of lymphocytes (Bouchareychas et al., 2020). ECs that activated CD137 signalling by an anti-CD137 antibody increased the expression of pAkt and NF-κB and induced an increase in IL6 in exosomes secreted by ECs. Furthermore, exosomes derived from CD137-modified ECs enhanced Th17 cell responses to promote plaque inflammation and stimulate EC dysfunction in AS (Xu et al., 2020a).

### Lipid Deposition

During AS, lipids gradually accumulate in the subendothelial space of impaired arteries, resulting in several lipid modification processes followed by macrophage and smooth muscle cell uptake in the arterial wall (Maguire et al., 2019). Exosomes, as transporters of proteins related to the modulation of this process, were reported to be secreted. CD36-containing exosomes derived from adipocytes mediated lipid uptake and HepG2 cell damage. Specifically, the study showed that exosomes derived from CD36-knockdown adipocytes were related to decreased lipid accumulation and apoptosis rate of HepG2 cells (Yan et al., 2021). However, brown adipose tissue-derived exosomes were proven to play a protective role in alleviating lipid accumulation in mice fed a HFD (Zhou et al., 2020).

circRNA_0046367 is an endogenous modulator of miR-34a that underlies hepatic steatosis. Its normalized expression promotes steatosis resolution by restoring the activity of peroxisome proliferator-activated receptor α (PPARα) and transcriptionally activating lipid metabolism-associated genes (Guo et al., 2017a). Overexpressed cirHIPK3 reduced the accumulation of lipids in HUVECs by activating autophagy, and both the reduction in lipids and activated autophagy led to attenuated AS. The stable knockdown of hsa_circH19 in human ADSCs upregulated the expression of lipogenesis-related genes and increased the number of lipid droplets formed (Zhu et al., 2020a). It was reported that circ_0075932 was highly expressed in adipose tissue but was found to be expressed at relatively low levels in most tissues. In obese patients and burned skin, hsa_circ_0075932 was significantly upregulated and was transmitted by exosomes from adipocytes to dermal keratinocytes to promote cell apoptosis and inflammation (Zhang et al., 2019d). However, the author did not explore the effect of this circRNA on lipid accumulation in the pathological progression in obese individuals.

EVs and circRNAs play various roles in the process of lipid metabolism and can provide therapeutic suggestions for interfering with lipid deposition and the progression of atherosclerosis.

### Formation of Foam Cells

Adipose tissue (AT) secretes hundreds of bioactive compounds, especially proinflammatory cytokines, and is recognized as an active endocrine organ. Exosomes isolated from visceral AT in HFD-induced obese mice significantly facilitated the generation of macrophage foam cells by downregulating ABCA1 and ABCG1 expression (Xie et al., 2018). In an early study, heat shock protein 27 (HSP27) was shown to be atheroprotective in a mouse model of AS but only in female mice. Macrophages secrete HSP27 via exosomes after stimulation with oestrogen (Rayner et al., 2009), and HSP27 reduces foam cell formation and atherogenesis by binding scavenger receptor-A on the surface of macrophages and reducing the uptake of acetylated LDL (ac-LDL) and inflammatory cytokine release (Rayner et al., 2008). Platelet-derived exosomes rapidly decreased the CD36 level in macrophages through enhanced ubiquitination and subsequent proteasome degradation, which reduced lipid load and interfered with the formation of foam cells (Srikanthan et al., 2014). Downregulation of CD36 attenuated lipid loading onto macrophages and the formation of foam cells. In other studies, exosomes derived from dendritic cells (Lin et al., 2021), adipocytes (Xie et al., 2018), HP-infected gastric epithelial cells (Yang et al., 2019) have been implicated in regulating macrophage foam cell formation to modulate AS process. In another study, EVs secreted by macrophage‐derived foam cells activated the ERK and Akt pathways and promoted migration and adhesion in VSMCs (Niu et al., 2016), which may be involved in crosstalk of the AS microenvironment.

According to the Gene Expression Omnibus database, in human THP-1 macrophages treated with ox-LDL (an *in vitro* AS model), 29 circRNAs were differentially expressed, as determined through linear models using the microarray data method. The study also predicted a circRNA/lncRNA-miRNA-mRNA network in ox-LDL-induced foam cells and indicated that the circRNAs were closely linked to foam cell formation (Wang et al., 2019d). In a recent study, the upregulation of circDENND1B significantly attenuated foam cell formation induced by ox-LDL by promoting cholesterol efflux (Xu et al., 2021a). In patients with a high coronary atherosclerotic burden, plasma levels of hsa_circ_0001445 are low. Furthermore, an *in vitro* test confirmed that hsa_circ_0001445 expression in human coronary SMCs and EVs is reduced under atherogenic lipoprotein conditions. (Vilades et al., 2020). Upregulation of circ-0029589 in ox-LDL-stimulated VSMCs promote the expression of insulin-like growth factor 2 and stromal interaction molecule and VSMC proliferation, migration and apoptosis (Liang et al., 2021). However, in ox-LDL-stimulated macrophages, circ-0029589 attenuated an increase in methylation levels, which contributed to the mechanism by which stromal interaction molecule 1 induced macrophage apoptosis during AS progression (Yu et al., 2020b; Guo et al., 2020; Huang et al., 2020). Further study of circ-0029589 expression levels in exosomes in the plasma or plaque microenvironment will reveal the crosstalk and functional mechanisms involved in the combined action of SMCs and macrophages.

### Vascular Classification

Vascular classification is a significant hallmark of AS. EV is associated with the initiation and progression of vascular calcification. Under pathological stimulation, macrophages and SMCs secrete EVs, which accumulate between collagen fibres and act as nucleation sites for ectopic mineralization of vascular walls, causing the formation of microcalcifications and macrocalcifications (Buffolo et al., 2022). It was reported that SMC-derived EVs initiate microcalcification in atherosclerotic plaques. Under hyperglycaemic conditions, S100A9 is upregulated and promotes macrophage release of calcific EVs, contributing to the formation of microcalcification within plaques (Kawakami et al., 2020).

Thousands of circRNAs have been detected in calcified human aortic valves, suggesting that they might be associated with vascular calcification (Chen et al., 2018b). Through analysing RNA sequencing and *in vitro* experimental data, circSamd4a has been found to be decreased in the VSMCs of rats and to have anti-calcification functions (Ryu et al., 2020). Melatonin contributes to ameliorating AS by counteracting the pyroptosis of ECs, inhibiting mitophagy activation and the NLRP3 inflammasome and regulating macrophage polarization to stabilize rupture-prone vulnerable plaques (Zhang et al., 2018b; Ma et al., 20182018; Ding et al., 2019). Furthermore, melatonin has been shown to reduce the level of circRIC3, contributing to ameliorate aortic valve calcification (Wang et al., 2020c). The circRNA TGFBR2 promotes aortic valve calcification via regulating osteoblast differentiation (Yu et al., 2021). Conversely, circSmoc1-2 has protective functions in vascular calcification by decreasing calcium deposition in VSMCs (Ryu et al., 2022). The association among EVs, circRNAs and vascular calcification should be taken into account because direct evidence of EV-circRNAs regulating vascular calcification is limited.

## EV-CircRNAs as Novel Biomarkers of AS

### Advantages of EV-CircRNAs as Biomarkers of AS

The clinical diagnosis of AS depends mainly on examination of images showing changes caused by arteriostenosis or plaque formation. Existing serum biomarkers, such as inflammatory cytokines (Hansson et al., 2006) and cholesterol and lipoprotein (Hoefer et al., 2015), lack specificity, and it is difficult to show different stages of AS, especially the early stage of AS.

Many circRNAs are abundantly expressed, some in a tissue- and developmental stage-specific manner, and numerous studies have shown significantly different expression levels between patients and healthy controls in many human diseases, such as aAS, Alzheimer’s disease, and malignant tumours. Hence, circRNAs have potential roles in noninvasive diagnosis and assessment in different disease stages (Jeck et al., 2013; Salzman et al., 2013; Hanan et al., 2017). Because of the novel characteristics and stability conferred by the covalently closed continuous loop structure and endonuclease resistance, circRNAs show intra- and extracellular stability and a long half-life (Jeck et al., 2013). CircRNAs are generally distributed in plasma (Zheng et al., 2017), urine (Chen et al., 2018a; Kölling et al., 2019), saliva (Bahn et al., 2015; Jafari Ghods, 2018) and cerebrospinal fluid (Li et al., 2020) secreted by tissue cells directly or packaged on EVs. Sumeng Wang et al. summarized the expression patterns of circRNAs in bodily fluids in multiple cancers and characterized their clinical application in liquid biopsy (Wang et al., 2021b). Li et al. first showed the existence of extensive circRNAs in exosomes, and the ratio of circRNA levels to linear RNA levels in exosomes was nearly 6-fold higher than the proportion in cells (Li et al., 2015). CircRNAs contained in EVs, especially in exosomes, are regarded as potential biomarkers and novel therapeutic targets in multiple cancers and immune and renal diseases (Jiang et al., 2020). These study results indicate that the expression pattern of EV-circRNAs may be significant in AS clinical diagnosis.

### Potential EV-CircRNAs as Biomarkers in AS

Serum levels of circR-284 were significantly elevated, while those of miR-221 were lower in acutely symptomatic patients than in asymptomatic patients. The ratio of serum circR-284: miR221 showed favourable sensitivity and specificity for use in detecting plaque rupture and stroke (Bazan et al., 2017). In addition, in coronary artery disease (CAD) patients, the expression levels of 795 circRNAs were found to be substantially different from those of controls (fold change [FC]>1.5), with 624 circRNAs upregulated and 171 circRNAs downregulated at a significant level (*p* < 0.05). Among these circRNAs, the area under the curve (AUC) of hsa_circ_0004104 and hsa_circ_0001879 combined with CAD risk factors and conventional markers (smoking and total cholesterol [TC] and serum creatinine levels) was 0.832 (95% CI, 0.788–0.876; *p* < 0.001), the sensitivity was 0.668 and the specificity was 0.890, demonstrating that these circRNAs can potentially advance the diagnosis and prognosis of CAD (Wang et al., 2019e). Hsa_circ_0001445 is stable in plasma samples, and its lower plasma levels in patients are accompanied by a higher coronary atherosclerotic burden, which leads to the identification of coronary AS in patients suspected of having stable CAD (Vilades et al., 2020). Circ-DLGAP4 downregulation in peripheral blood mononuclear cells (PBMCs) of acute ischaemic stroke (AIS) patients showed promise for predicting the risk and severity of AIS. The study suggested that PBMC circ-DLGAP4 was negatively associated with inflammation levels in AIS (Zhu et al., 2019b). Studying patients with large-artery AS (LAA)-type AIS, Wangtao Zhang et al. identified 182 upregulated and 176 downregulated circRNAs and identified hsa_circRNA_0001599, with sensitivity and specificity values of 64.41 and 89.93%, as a putative circRNA biomarker for diagnosis (Li et al., 2021b).

Since they are enveloped by bilayer lipid membranes, circRNAs can be stably transferred through bodily fluid over long distances. The detection of tissue-specific markers on EV membranes helps to distinguish the sources of circRNAs, which are produced by multiple tissue cells. This possibility suggests that circRNAs enriched in EVs may contain information to help with diagnosis and prognosis. Compared to non-AS patients, the circ_0003204 level in EVs obtained from cerebral AS patients was markedly higher, which indicated a higher risk of life-ending events, and HAECs exposed to ox-LDL exhibited a similar tendency. Significantly, the AUC of the combination of circ_0003204 in plasma EVs and LDL cholesterol (LDL-C) levels used for predicting cerebral AS was 0.875, demonstrating its potential diagnostic value in cerebral AS (Zhang et al., 2020c). Circ_DLGAP4, which is involved in AIS, was recently found to be increased in exosomes isolated from patients with diabetic kidney disease, who have a high risk of developing cardiovascular disease (Bai et al., 2020a). Numerous studies have been concerned with the involvement of EVs in atherogenesis and atheroprotection (Peng et al., 2020b). CircRNAs, as important cargoes enriched in EVs, have great diagnostic and prognostic potential in AS, but relevant research has been limited to date.

## Therapeutic Potential of EV-CircRNAs in AS

### EV-CircRNAs Provide a Novel Therapeutic Target for AS

Numerous studies have found that exosomal circRNAs play a major part in crosstalk in the tumour microenvironment. Exosomal transfer of circRNA influences proliferation, apoptosis, migration (Wu et al., 2020), the malignant phenotype (Fang et al., 2020), the EMT (Xu et al., 2021b) and chemoresistance (Xu et al., 2021c) of tumour cells to promote or inhibit tumour invasion and metastasis. Furthermore, exosomal circRNA obtained from tumour cells enhanced circRNA expression in surrounding normal cells and stimulated EMT progression (Fang et al., 2020). CircFNDC3B and circFNDC3B-enriched exosomes inhibited the angiogenic properties of colorectal cancer (Zeng et al., 2020). Many transmitted circRNAs have been proven to play a tumour-promoting role in cancer and may act as novel therapeutic targets for the therapy in malignant tumours. For example, CircEhmt1 was upregulated in pericytes induced by hypoxic conditions and was transferred from pericytes to endotheliocytes via exosomes, which may be a protective mechanism against high glucose-induced injury conferred by circEhmt1 overexpression (Ye et al., 2021). EV-circRNAs can participate in proatherogenic or antiatherogenic processes by acting on ECs injury, lipid accumulation, vesicular inflammation, and the phenotypic transition of SMCs. Thus, interfering with these functions of circRNAs may be a novel therapeutic target for AS.

### EV-CircRNAs From Modified Cells for Use in Novel Therapeutic Strategies for AS

According to their long half-life, low immunogenicity and capacity to cross biological barriers, EVs, especially exosomes, have been novel hotspots in therapeutic research because of their great potential as “natural nanoparticles” that can deliver therapeutic substances (Jiang and Gao, 2017). Exosomes derived from circAkap7-modified adipose-derived mesenchymal stem cells show therapeutic effects on cerebral ischaemic injury, as proven in a mouse model of transient middle cerebral artery occlusion (tMCAO). The study showed that Exo-circAkap7 treatment reduced the infarct volume and tMCAO-induced sensorimotor dysfunctions by attenuating cerebral apoptosis. Further study revealed that the protective mechanism conferred by Exo-circAkap7 involves the promotion of ATG12-mediated autophagy and NRF-mediated oxidative stress and inflammatory responses to oxygen- and glucose-deprivation/reoxygenation induction in primary astrocytes cocultured with exo-circAkap7 *in vitro* (Xu et al., 2020b). Early studies have indicated that endothelial progenitor cells (EPCs) play significant roles in atherogenesis by mobilizing EPCs in bone marrow and mediating the regeneration of ECs to repair injured sites (Condon et al., 2004; Hunting et al., 2005). Rongfeng Shi also found that exosomes derived from mmu_circ_0000250-modified ADSCs restored the function of vascular EPCs under high-glucose conditions by activating autophagy, an outcome validated by evidence of a reverse effect induced by treatment with the autophagy inhibitor chloroquine (CQ). In EPCs, mmu_circ_0000250 elevated the expression of the SIRT1 upstream modulator of autophagy via miR-128 absorption (Shi et al., 2020). Ischaemic stroke is the primary cause of permanent disability and mortality in numerous types of AS. We developed a technology that successfully delivered stroke patient-implicated circSMCH1 to the brain through engineered EVs, and the administration of circSMCH1 significantly improved functional recovery after stroke by enhancing neuroplasticity and inhibiting glial reactivity and peripheral immune cell infiltration (Yang et al., 2020b). Exosomes derived from ischaemic-preconditioned astrocyte-conditioned medium (IPAS-CM) exerted neuroprotection. CircSHOC2 in IPAS-EXOs ameliorated neuronal apoptosis and suppressed neuronal injury by regulating autophagy through modulating the miR-7670–3p/SIRT1 axis (Chen et al., 2020b). These studies provide new insight into therapeutic strategies for stroke treatment. Modified EV-circRNAs can significantly influence the physiological and pathological pathways in AS, providing a novel therapeutic strategy for AS.

With the characteristics of high specificity and low immunogenicity, EVs show good prospects for use in the precise treatment of AS lesions and other applications. The combination of exosomes and circRNAs is a very promising treatment strategy in the near future.

## Conclusion and Future Perspectives

Over the past few decades, our understanding of the classical risk factors for atherosclerotic progression has improved rapidly. The molecular mechanism by which these factors lead to AS development has been largely clarified. In recent years, circRNAs in EVs have become a new hot topic in human disease research. EVs were found to be important immune transporters in metabolic diseases and AS. Under the pathological conditions of high glucose, abnormal lipid levels and/or high inflammatory cytokine levels, EVs, the crucial mediators of intracellular communication, serve as cellular function modulators or products of cellular function modulation.

CircRNAs, as the main cargoes in EVs, have been proven to be significant in AS progression. The following interaction between proatherogenic factors and EV-circRNAs has been established: circRNAs modulate proatherogenic factors, and antiatherogenic factors change the level or variety of circRNAs in EVs. Because of their stability, specificity, significant differences in expression and multiple functions, EV-circRNAs show great potential as reliable biomarkers and contributors to therapeutic strategies in different stages of AS.

Considering the studies explored in this review, proatherogenic factors can change circRNA levels in AS-associated cells by modulating the packaging of circRNAs in EVs during AS pathogenesis. Taken together, the cells involved in the progression of AS such as ECs, macrophages, and SMCs have the potential to become donor cells based on different stimuli, while EV-cirRNAs are involved in the regulation of the interaction between cells, resulting in the destruction of the endothelial cell barrier, macrophage and smooth muscle cell foaming, and smooth muscle cell phenotype transformation, etc., are involved in the progression of atherosclerosis. The mechanism by which donor cells release EV-cirRNA needs to be further explored.

On the basis of this phenomenon, the development of EV-circRNAs to modulate various signalling pathways in anti-AS processes may be a promising approach. However, direct research on the effects of EV-circRNAs on AS development is still insufficient to clarify the exact molecular mechanism involved in the effects of EV-circRNAs and proatherogenic factors on AS. Additional investigation of the predictive roles and functions of EV-circRNAs in AS will lead to a better understanding of the pathophysiological and physiological processes of AS and will hopefully lead to intense research in this area.
